# Mo-derived perivascular macrophage recruitment protects against endothelial cell death in retinal vein occlusion

**DOI:** 10.1186/s12974-019-1547-8

**Published:** 2019-07-27

**Authors:** Christophe Roubeix, Elisa Dominguez, William Raoul, Xavier Guillonneau, Michel Paques, José-Alain Sahel, Florian Sennlaub

**Affiliations:** 1 INSERM, CNRS, Institut de la Vision, 17 rue Moreau, Sorbonne Université, UPMC Univ Paris 06, F-75012 Paris, France; 2 Université François Rabelais de Tours, CNRS, GICC UMR 7292, Tours, France; 3 Centre Hospitalier National d’Ophtalmologie des Quinze-Vingts, INSERM-DHOS CIC 1423, F-75012 Paris, France; 4 Berlin Institute of Health, Berlin, Germany

**Keywords:** BRVO, Perivascular macrophages, CCR2, Endothelial cells

## Abstract

**Background:**

To decipher the role of monocyte-derived macrophages (Mφs) in vascular remodeling of the occluded vein following experimental branch retinal vein occlusion (BRVO).

**Methods:**

The inflammation induced by laser-induced BRVO on mice retina was evaluated at different time points by RT-PCR looking at inflammatory markers mRNA level expression, Icam-1, Cd11b, F4/80, Ccl2, and Ccr2 and by quantification of Iba1-positive macrophage (Mφ) density on Iba1-stained retinal flatmount. Repeated intraperitoneal EdU injection combined with liposome clodronate-induced monocyte (Mo) depletion in wildtype mice was used to differentiate Mo-derived Mφs from resident Mφs. Liposome clodronate Mo-depleted wildtype mice and Ccr2-deficient mice were used to evaluate the role of all CCR2^+^ and CCR2^neg^ Mo-derived Mφs on EC apoptosis in the occluded vein.

**Results:**

cd11b, ICAM-1, F4/80, Ccl2, and Ccr2 mRNA expression were increased 1, 3, and 7 days after vein occlusion. The number of parenchymal (parMφs) and perivascular (vasMφs) macrophages was increased 3 and 7 days after BRVO. The systemic depletion of all circulating Mos decreased significantly the BRVO-induced parMφs and vasMφs macrophage accumulation, while the deletion of CCR2^+^-inflammatory Mo only diminished the accumulation of parMφs, but not vasMφs. Finally, apoptotic ECs of the vein were more numerous in fully depleted, liposome clodronate-treated mice, than in Ccr2^−/−^ mice that only lack the recruitment of CCR2^+^ inflammatory Mos.

**Conclusions:**

BRVO triggers the recruitment of blood-derived parMφs and vasMφs. Interestingly, vasMφs accumulation was independent of CCR2. The observation that the inhibition of the recruitment of all infiltrating Mφs increases the vein EC apoptosis, while CCR2 deficiency does not, demonstrates that CCR2^neg^ Mo-derived vasMφs protect the ECs against apoptosis in the occluded vein.

## Introduction

Branch retinal vein occlusion (BRVO) leads to extensive vascular remodeling and is an important cause of visual impairment due to vascular leakage and retinal edema. We have previously shown that experimental BRVO is associated with an acute wave of endothelial cell (EC) apoptosis of the occluded vein and the upstream capillaries, extravasation, and retinal edema. In this model, ECs subsequently proliferate and the edema resolves [1]. BRVO has also been shown to be associated with increased levels of chemokines and cytokines (including CCL2, which induces inflammatory monocytes (Mos) recruitment) in experimental BRVO [1, 2] in aqueous [3, 4] and in vitreous humor [5–7] of BRVO patients. In experimental BRVO, resident Iba1+ macrophages (rMφs) are activated and blood-derived Mφs (iMφs) are recruited [2] in the retina but their role in vascular remodeling in BRVO remains unknown. Mononuclear phagocytes (MPs) are a family of cells that include resident macrophages (rMφ), such as microglial cells (MCs) and perivascular Mφs (vasMφs) in the retina, and Mo-derived Mφs (iMφs) that are recruited to the injured tissue upon chemokine induction. Blood monocytes can be divided into two subsets [8–10]: the CCR2^+^ “inflammatory” monocytes, the blood-born precursors of inflammatory macrophages and inflammatory dendritic cells, and the CCR2^−^ “non-inflammatory” monocytes that patrol the vasculature [10, 11] but also participate in the inflammatory reaction in certain affections, such as atherosclerosis [12]. Using the laser-induced BRVO model in mice, we here confirm that Mo-derived Mφs are recruited to the occluded retina. Using *Ccr2*-deficient mice and clodronate-induced circulating Mos depletion, we identified two major subset of infiltrating MPs: 1/parenchymal Mφs (parMφs) and 2/vasMφs that were recruited independently of CCL2/CCR2 signaling. Mos depletion, but not CCR2 depletion, significantly exaggerated the wave of BRVO-induced EC apoptosis, suggesting that Mo-derived CCR2-negative vasMφs protect EC in BRVO against apoptosis.

## Material and methods

### Animals

Eight to 12-weeks-old C57BL/6JRj male mice were purchased from Janvier SA (Le Genest-Saint-Isle, France). *Ccr2*^*RFP/RFP*^ (B6.129(Cg)-*Ccr2*^*tm2.1Ifc*^/J) mice were purchased from Jackson Laboratory (Bar Harbor, USA). Mice were maintained at the Institut de la Vision animal facility under pathogen-free conditions. All animals were housed in a 12-h/12-h light/dark cycle with food and water available ad libitum.

### Ethics statement

All manipulations were performed in accordance with the association for research in vision and ophthalmology (ARVO) Statement for the Use of Animals in Ophthalmic and Vision Research. In addition, all the experimental procedures were permitted by the Institutional Animal Care and Use Committee, “Comité d’éthique pour l’expérimentation animale Charles Darwin” (ID Ce5/2012/013), which also specifically approved the study reported in the present manuscript.

### BRVO model

Occlusion of one branch of the retinal vein was performed by laser photocoagulation as previously described [1, 13] with slight modifications. Pupils were dilated with tropicamide (Mydriaticum, Théa, France) and phenylephrin (Neosynephrine, Europhta, France). Animals were injected with 100 μl of 1% fluorescein (FluoresceineSodique Faure 10%, Novartis Pharma, France) for dye enhancement and were immediately anesthetized by inhalation of isoflurane (Axience, France). Fundus was visualized with a slit lamp (BQ 900, Hagg-Streitt, Swiss) through a fundus laser lens (OFA 2.0 mm, Ocular Instruments Inc., USA) positioned on the mouse cornea with eye gel (Lubrithal, DechraVetxx, France). Seven to 12 laser impacts (0.5 s, 200 mW, 50 μm spot size, Laser Yag 532 Eyelite (Alcon, USA)) were performed on a superior vein, 2 to 3 disc diameter from the optic disc. The complete and the persistence of the occlusion were checked using fluorescent angiography with Micron III device (Phoenix Labs). Development of an eventual recanalization of the vein was checked during and at the end of the study by a control fundus.

Laser impacts were performed on retina away from any veins to exclude consequence of the laser alone. Mice in which retinal hemorrhage occurred or with incomplete occlusion were excluded from the study.

### RT-PCR

At different times after BRVO, part of retina corresponding to the occluded area was carefully dissected in RNase-free conditions. Total RNA was isolated with Nucleospin RNAII (Macherey Nagel, France). Single-stranded cDNA was synthesized from total RNA (pretreated with DNaseI amplification grade) using oligo-dT as primer and superscript II reverse transcriptase (Life Technologies, France). Subsequent real-time polymerase chain reaction (RT-PCR) was performed using cDNA, Taqman, or SYBRgreen Gene Expression Master Mix (Life Technologies) and primers (0.5 pmol/μl) available upon request. Results were normalized by expression of S26. PCR reactions were performed in 45 cycles of 15 s at 95 °C and 45 s at 60 °C. Primers used during the study are listed below.

**Table:** 

S26_AS	AGCTCTGAATCGTGGTG
S26_S	AAGTTTGTCATTCGGAACATT
CCL2_AS	ACCCATTCCTTCTTGGGGTC
CCL2_S	GATGCAGTTAACGCCCCACT
CCR2_AS	GGCATGAGGCTGTCAG
CCR2_S	ATTGTCCATGTTGTCATAGAT
ICAM1_AS	CAGGGTGAGGTCCTTGCCTA
ICAM1_S	TCCGCTACCATCACCGTGTA
F4/80_AS	TGGAAGTGGATGGCATAGATG
F4/80_S	TTCACTGTCTGCTCAACG
CD11b_AS	ATGCTGTGCTGTCCTCTCTG
CD11b_S	ATCTCTGCTGGCTTTCCAGT

### Immunohistochemistry

Mice were killed by CO_2_ inhalation, and the eyes were enucleated and fixed in 4% paraformaldehyde for 30 min at room temperature. After several washes in PBS, the cornea and lens were removed and the retina was carefully detached from RPE/choroid/sclera. The retinas were incubated overnight with primary antibodies (goat anti-collagen IV, 1:100, Serotec, USA; rabbit anti-Iba1, 1:400, 1:500, Millipore, USA) in PBS supplemented with 0.5% Triton X-100, followed by incubation with appropriate Alexa-coupled secondary antibodies (Life Technologies). The retinas were flatmounted and viewed with a fluorescence microscope (DM5500B, Leica, France) or with a confocal microscope (FV1000, Olympus, France).

All cell counting was performed in the inner retina (GCL/IPL). Vessel lengths and vascular areas were calculated with the “angiogenesis tube formation” add-in [14] available on MetaMorph 2D software (Molecular Devices, France).

### MP tracing by systemic EdU injection and Mo depletion with clodronate liposomes

Repeated systemic EdU injections are able to stain monocytes in bone marrow as previously described [15]. With this procedure, 76% of CD115+ circulating monocytes showed EdU incorporation (during 4 days). Briefly, mice were treated daily by three intraperitoneal injections of EdU (50 mg/kg, Life Technologies) 1 day before BRVO and in the three following days [1]. Intraretinal proliferation was measured traced by intravitreous injections of EdU (6.7 μg/μl, 2 μl/eye). Additionally, mice were intravenously injected with 100 μl of control liposomes (placebo control liposomes for clophosome-A) or liposomes containing clodronate (clophosome-A-anionic liposomal clodronate for macrophage depletion, FormuMax) the day of occlusion and the two following days leading to a decrease of circulation monocyte population of 84% [15]. The eyes were removed, and retinal flatmounts were labeled with Iba1 and revealed for EdU incorporation (Click-iT EdU Imaging Kit, Life Technologies) for the quantification of Iba1+ and EdU+ cells.

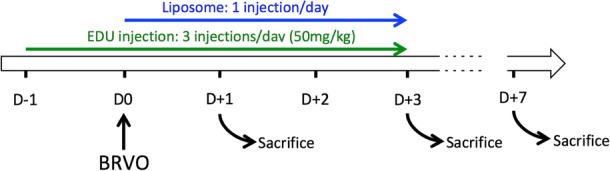



### Terminal deoxynucleotidyl transferased UTP nick end labeling

After immunohistochemistry, retinal flatmounts were pretreated with frozen methanol for 20 min and then frozen methanol/acetic acid (2:1) for another 20 min. After washing with PBS, flatmounts were incubated overnight at 4 °C with the reaction mixture as described by the manufacturer’s protocol (In Situ Cell Death Detection Kit, Roche Diagnostics, USA) and then for 90 min at 37 °C. After reaction was stopped by washing with PBS at RT, nuclei were counterstained with Hoechst (Sigma-Aldrich, France). The retinas were mounted, viewed, and photographed with a fluorescence microscope (DM5500B, Leica) or with a confocal microscope (FV1000, Olympus).

### Statistical analysis

GraphPad Prism 5 (GraphPad Software, San Diego, USA) was used for data analysis and graphic representation. All values are reported as mean ± SEM. Statistical analysis is described in the legend of each figure. *P <* 0.05 was considered statistically significant.

## Results

### BRVO increases the number of perivascular and parenchymal mononuclear phagocytes

**Fig. 1:**
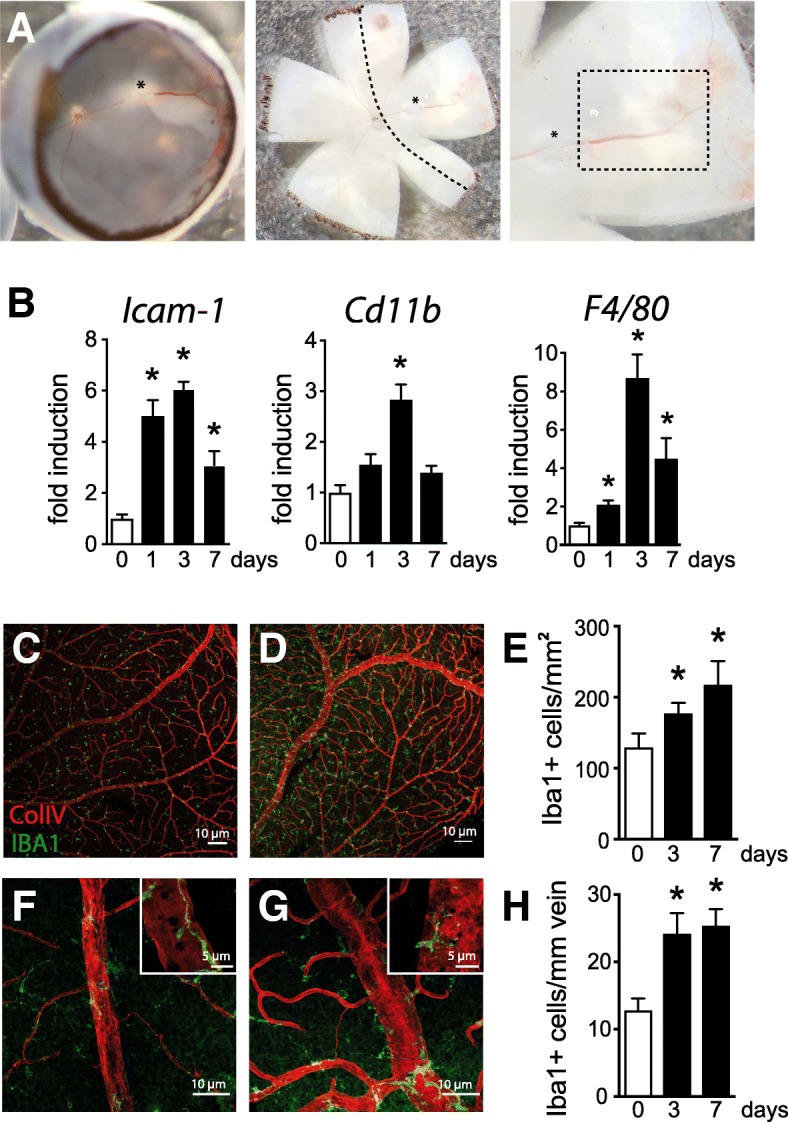
BRVO increases the number of perivascular and parenchymal mononuclear phagocytes. Photographs of the eyecup and retinal flatmount of the retina with the visible vein occlusion site (*), dilated vein, and hemorrhages at day 1 after BRVO. Dotted line shows the dissected area of the retina used for RT-PCR and the dotted rectangle the area where perivascular Mφs were counted during the study (**a**). *Icam-1*, *cd11b*, and *F4/80* real-time RT-PCRs in the occluded retina at indicated time points. The results were normalized to S26 expression. Values in histograms are mean ± SEM of mRNA expression of occluded area from 4 eyes per group (**b**, *n* = 6, Mann-Whitney *t* test; Icam-1: *P = 0.0095 day 0 versus day 1, *P = 0.0047 day 0 versus day 3, and *P = 0.0095 day 0 versus day 7; Cd11b: *P = 0.0095 day 0 versus day 3; F4/80: *P = 0.0424 day 0 versus day 1, *P = 0.0047 day 0 versus day 3, and *P = 0.0095 day 0 versus day 7). Representative images of the inner retina of Iba1 (green) and ColIV (red) double-labeled retinal flatmounts of control (**c**) and occluded vein area 3 days after occlusion (**d**). Magnification on control vein in the upstream retina (**f**) and on the occluded vein (**g**) 3 days after occlusion. Quantification of the parenchymal Iba1^+^ Mφs per square millimeter (**e**, Mann-Whitney *t* test, Iba1^+^ cells/mm of vein: day 0 versus d3day *P = 0.0229 and day 0 versus day 7 *P = 0.0424) and the number of perivascular Iba1^+^ Mφs per millimeter of vein (**h**, Mann-Whitney *t* test, Iba1^+^ cells/mm^2^: day 0 versus day 3 *P = 0.0229 and day 0 versus day 7 *P = 0.0424) of control retinas and in the occluded vein 3 and 7 days after the occlusion. Icam-1, intracellular adhesion molecule 1; Iba1, ionized calcium-binding adapter molecule 1. Scale bar **c** and **d** = 10 μm. Inset = 5 μm. Scale bar **e** and **f** = 50 μm

**Fig. 2:**
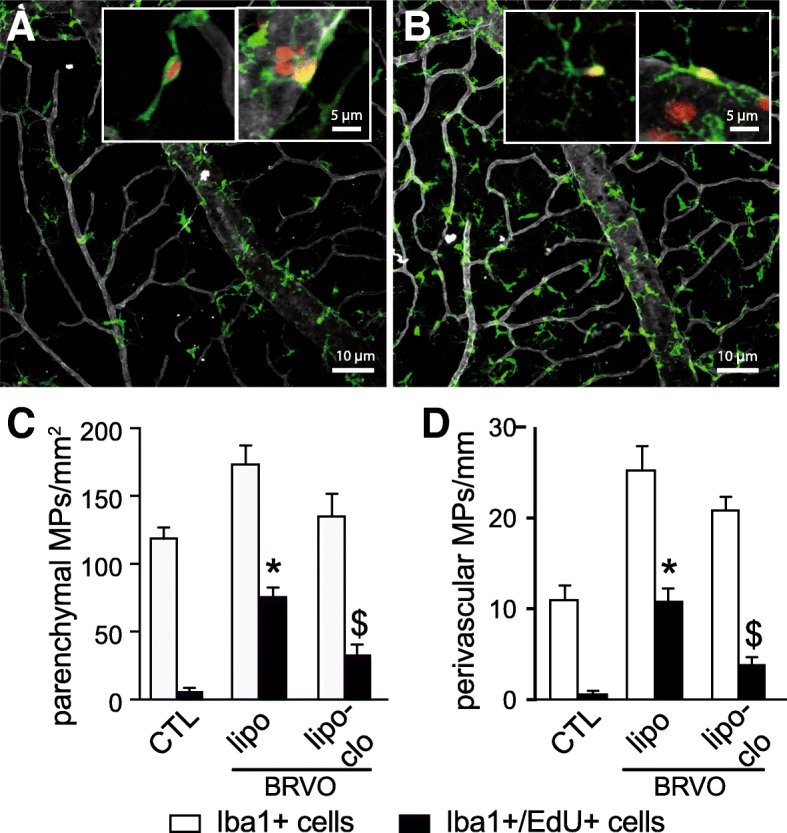
The increased population of Iba-1+ cells in the inner retina after BRVO is partly due to blood-derived macrophage infiltration. Representative images of the inner aspect of Iba1 (green), ColIV (white), and EdU (red) triple-labeled retina of control animals (**a**) and 3 days after BRVO (**b**) that were intraperitoneally injected with EdU for 4 days. The insets depict representative EdU+Iba1+ parenchymal (left panel) and perivascular (right panel) Mφs for each condition at higher magnification as they are not visible in the overview. Quantification of Iba1^+^ Mφs (white columns) and Iba1^+^EdU^+^Mφs (black columns) of the parenchymal Mφs per square millimeter (**c**, *n* = 6/group, Mann-Whitney *t* test, control parenchymal Iba1^+^EdU^+^Mφs versus BRVO liposome parenchymal Iba1^+^EdU^+^Mφs *P = 0.0143; BRVO liposome parenchymal Iba1^+^EdU^+^Mφs versus BRVO liposome clodronate parenchymal Iba1^+^EdU^+^Mφs ^$^*P* = 0.0143) and the number of perivascular Iba1^+^ Mφs per millimeter of vein (**d**, *n* = 6/group, Mann-Whitney *t* test, control perivascular Iba1^+^EdU^+^Mφs versus BRVO liposome parenchymal Iba1^+^EdU^+^Mφs *P = 0.0143; BRVO liposome parenchymal Iba1^+^EdU^+^Mφs versus BRVO liposome clodronate perivascular Iba1^+^EdU^+^Mφs ^$^*P =* 0.0095) of control retinas and retinas from BRVO mice receiving intravenous control liposome injections (lipo) or clodronate liposome injections (lipo-clo). BRVO, branch retinal vein occlusion; lipo, control liposome-treated; lipo-clo, liposome clodronate-treated; Iba1, ionized calcium-binding adapter molecule 1. Scale bar **a** and **b** = 10 μm. Inset = 5 μm

To better characterize the inflammatory processes in BRVO, we first quantified by RT-PCR the mRNA expression of intercellular adhesion molecule 1 (*Icam-1)*, involved in leukocyte diapedesis, and the mononuclear phagocyte (MP) markers *Cd11b* and *F4/80* at day 1, day 3, and day 7. The upstream retinas of the occluded veins are easily recognizable by the dilated vein and the diffused retinal extravasations. These areas were carefully dissected in RNase-free conditions (Fig. 1a). All three transcripts were strongly and significantly induced at all investigated time points compared to non-occluded (white bar—day 0), peaking at day 3 (Fig. 1b). Analysis at day 3 and day 7 of Iba1 (green staining) and collagen IV (ColIV, red staining) double-labeled retinal flatmounts on the occluded area (rectangle—Fig. 1a) revealed two distinct populations of MPs in the inner retina, parenchymal and perivascular MPs. Vascular Mφs were distinguished from parenchymal Mφs by their more rounded and elongated cell bodies (Fig. 2b inset) and their physical contact with the ColIV+ retinal vein. The density of parenchymal macrophages (parMφs), located in the ganglion cell layer with no direct contact to the vasculature, increased by 46.9% at day 3 (190.1 ± 16.3 cells/mm^2^) and + 68.3% at day 7 (217.8 ± 36.7 cells/mm^2^) compared to non-occluded retina (129.4 ± 24.0 cells/mm^2^; Fig. 1c–e). Similarly, the number of elongated, ramified perivascular macrophages (vasMφs) located around the occluded vein appeared more numerous compared to non-occluded retina (Fig. 1f, g) [16]. Cell counts of vasMφs per vein length at day 3 (24.22 ± 3.8 cells/mm) and at day 7 (25.37 ± 3.5 cells/mm) revealed a nearly twofold significant increase of vasMφs compared to non-occluded retina (12.81 ± 2.1 cells/mm) at both time points (Fig. 1h).

Together our analysis shows that the number of both, vasMφs and parMφs, significantly increase after BRVO at 3 days and for the whole of the observation period.

### The increased population of Iba-1+ cells in the inner retina after BRVO is partly due to blood-derived macrophage infiltration

To evaluate the participation of monocyte recruitment from the bloodstream versus resident Mφs and MCs in BRVO-induced MP accumulation at day 3, we injected mice three times a day for 4 days (from 24 h prior to occlusion to day 3) intraperitoneally with the traceable nucleotide EdU, which we showed marks 75% of circulating CD115^+^ monocytes, but not resident retinal MCs and Mφs [15]. Furthermore, we inhibited the accumulation of Mo-derived Mφs by additional daily intravenous (day 0 to day 2) of either empty control liposomes or clodronate-containing liposomes, which we showed to lower the number of circulating monocytes by at least 85% for 24 h [15].

Micrographs of retinal areas of the mid-periphery of EdU (red staining), Iba-1 (green staining), and ColIV (white staining) triple-stained retinal flatmounts of non-occluded eyes from mice that were repeatedly injected with EdU for 4 days without liposome injection (CTL—Fig. 2a) and of the occluded vein of 3 days post-BRVO mice (BRVO—Fig. 2b) confirm the accumulation of Iba1^+^parMφs and vasMφs upstream of the vein occlusion and surrounding retina described above. EdU^+^Iba1^+^ parMφs and vasMφs (inset Fig. 2a, b) were visible in both occluded and non-occluded retina. However, quantification of EdU^+^Iba1^+^ and EdU^−^Iba1^+^ parMφs (Fig. 2c) and vasMφs (Fig. 2d) revealed that EdU^+^Iba1^+^ Mφs are rare in the non-occluded control eyes (CTL). Control liposome-injected experimental BRVO mice revealed that around half of the Iba1^+^ parMφs and vasMφs were EdU positive. This could be due either to the recruitment of EdU^+^Mo that differentiate into EdU^+^Iba1^+^Mφs or to EdU incorporation into locally proliferating Mφs. To differentiate the two, we depleted the mice of their circulating Mos by intravenous liposome clodronate injections, which very significantly reduced the numbers of EdU^+^Iba1^+^ parMφs and vasMφs in both populations. The Iba1^+^EdU^neg^ cells (white bars minus the black bars) stayed stable suggesting that they were not affected by the intravenous injection of clodronate liposomes.

In conclusion, our data demonstrates that parMφs and vasMφs that accumulate after BRVO are at least in part derived from Mo and that the accumulation of Mo-derived parMφs and vasMφs can efficiently be inhibited by Mo depletion in experimental BRVO.

### CCR2^+^Mo-derived Mφ participates in parMφs but not in vasMφs accumulation after vein occlusion

**Fig. 3:**
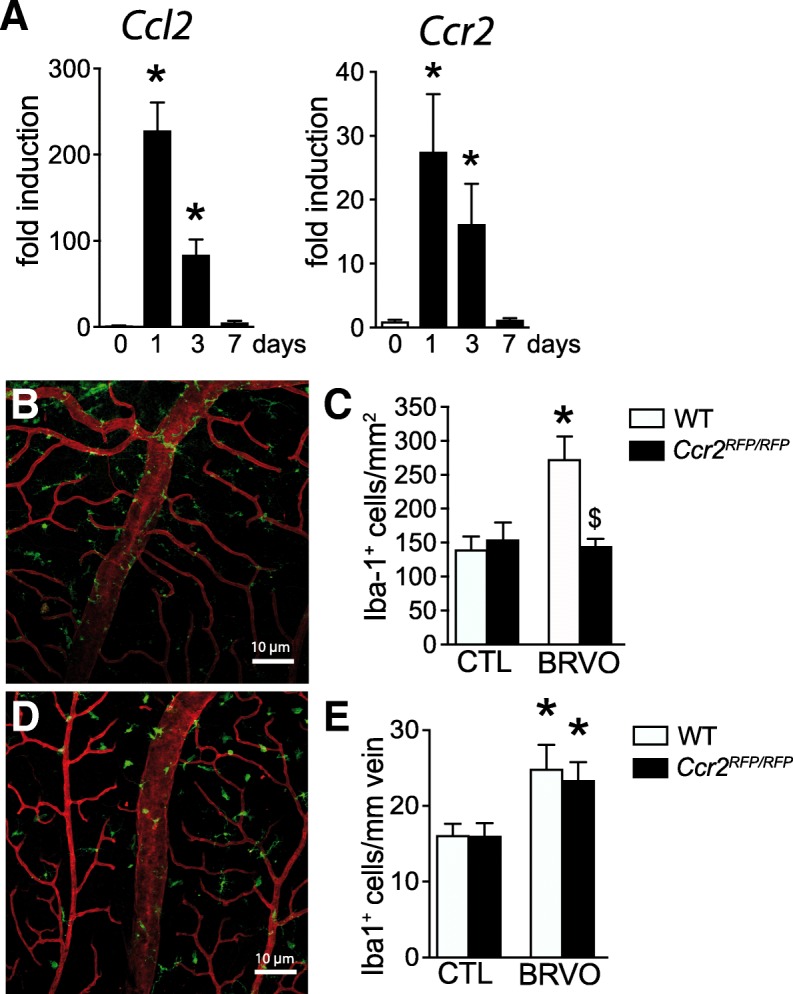
CCR2^+^Mo-derived Mφ participates in parMφs but not vasMφs after vein occlusion. *Ccl2* and *Ccr2* real-time RT-PCRs in the occluded retina at indicated time points (**a**). The results were normalized to S26 expression. Values in histograms are the mean ± SEM of mRNA expression on the occluded area from 4 eyes per group (**a**; *n* = 4/group, Mann-Whitney *t* test, *Ccl2* and *Ccr2* expression at day 0 versus day 1 and versus day 3, *P = 0.0286). Representative images of the inner retina of Iba1 (green) and ColIV (red) double-labeled retinal flatmounts of occluded vein in WT (**b**) and in *Ccr2*^*GFP/GFP*^ (**d**) mice 3 days after vein occlusion. Quantification of the parenchymal Iba1^+^ Mφs per square millimeter (**c**; *n* = 4 WT and *n* = 6 *Ccr2* ^*GFP/GFP*^, Mann-Whitney *t* test, Iba1^+^ cells/mm^2^ in control versus BRVO *P = 0.0143; BRVO in WT versus in *Ccr2*^*GFP/GFP*^ mice ^$^*P =* 0.0246) and the number of perivascular Iba1^+^ Mφs per millimeter of vein (**e**, iba1^+^ cells/mm vein in control versus BRVO for WT, *P = 0,0143, and *Ccr2*^*GFP/GFP*^ mice, *P = 0.0095) of control retinas and in the occluded vein 3 days after the occlusion. Ccl2, chemokine ligand 2; Ccr2, C-C chemokine receptor type 2; BRVO, branch retinal vein occlusion; Iba1, ionized calcium-binding adapter molecule 1. Scale bar **b** and **d** = 10 μm. Inset = 5 μm

CCL2 strongly participates in the recruitment of CCR2^+^ monocytes, which differentiate in the tissue into inflammatory Mφs during the early phases of the inflammatory response [8–10]. Quantification of mRNA by RT-PCR revealed a strong induction of Ccl2 and Ccr2 at day 1 and day 3 before falling back to basal expression levels at 7 days following the vein occlusion. To investigate the participation of CCR2^+^ Mos in the accumulation of Mo-derived parMφs and vasMφs, we submitted wildtype and *Ccr2*^*RFP/RFP*^ mice to BRVO and quantified Mφs on Iba1 (green staining) and CollIV (red staining) double-labeled retinal flatmounts. We previously demonstrated that red fluorescent protein (RFP) expression cannot be used as a marker to determine whether a Mφ is derived from a CCR2^+^ monocyte as the Ccr2 promotor activity that drives the RFP expression in *Ccr*^*RFP/RFP*^ mice quickly comes to a halt after tissue infiltration [15]. The BRVO-induced increase in a number of parMφs (Fig. 3b, c) observed in wildtype mice was significantly reduced in *Ccr2*^*RFP/RFP*^ mice presenting the same level of parMφs than in non-occluded retina (CTL). Interestingly, Ccr2 deficiency had no effect on the accumulation of vasMφs (Fig. 3d, e) after BRVO.

These results suggested that contrary to the increase of parMφs, increased numbers of vasMφs are not derived from CCR2^+^ monocytes.

### Mo-derived perivascular Mφs inhibit endothelial cell apoptosis

**Fig. 4:**
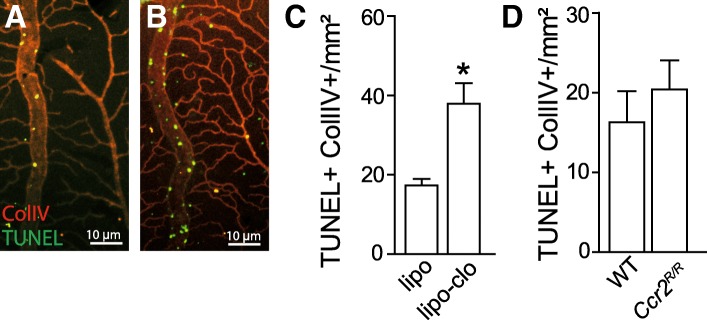
Mo-derived perivascular Mφs inhibit endothelial cell apoptosis. Representative images of the inner aspect of the of ColIV (red) and TUNEL (green) double staining of the occluded vein area 3 days after the occlusion in control liposome (**a**) and clodronate liposome (**b**)-treated mice. Quantification of ColIV+TUNEL+ cells on the occluded area and comparison between control liposome and clodronate liposome-injected mice (**c**, *n* = 8, Mann-Whitney *t* test, *P = 0.0004) and between WT and Ccr2^*GFP/GFP*^ mice (**d**). lipo, control liposome-treated; lipo-clo, liposome clodronate-treated; ColIV, collagen IV; TUNEL, terminal deoxynucleotidyl tranferase dUTP nick end labeling. Scale bar **a** and **b** = 10 μm

We have previously showed that ECs of the occluded vein undergo a wave of TUNEL+ apoptosis during the first 3 days following the obstruction followed by EC proliferation that mainly occurs thereafter [1]. TUNEL (green staining) and ColIV (red staining) double labeling of retinal flatmounts 3 days after experimental vein occlusion revealed TUNEL^+^ColIV^+^ apoptotic ECs on occluded veins of control liposome-treated mice (Fig. 4a) as previously described for untreated animals [1]. However, TUNEL^+^ColIV^+^ apoptotic ECs appeared more numerous in liposome clodronate-treated mice (Fig. 4b), which lack the Mo-derived vasMφs accumulation (Fig. 2). Interestingly, quantification of the number of TUNEL^+^ColIV^+^ apoptotic ECs upstream of the vein occlusion was significantly increased in liposome clodronate-treated mice (Fig. 4c) but showed no difference between the wildtype and *Ccr2*^*RFP/RFP*^ mice (Fig. 4d).

Taken together, as others and we previously showed that intravenous liposome clodronate [17, 18] does not directly induce EC apoptosis, our data suggests that the recruitment of CCR2^neg^Mo-derived vasMφs protects EC of the occluded vein from apoptosis.

## Discussion

We have previously shown that experimental BRVO is associated with an acute wave of endothelial cell (EC) apoptosis of the occluded vein and the upstream capillaries followed by the subsequent proliferation of the ECs [1]. We here show that experimental BRVO is accompanied by an increased expression of Icam-1 (that plays a central role in leukocyte recruitment to an injured tissue [19]), CD11b (an integrin that is more strongly expressed in Mo than MCs; Immgen.org), and F4/80 (EMR1, strongly expressed in macrophages during inflammation). Indeed, we demonstrate that Iba1^+^Mφs accumulate after BRVO and we identified two subtypes of Mo-derived Mφs: parMφs, accumulating in the inner retinal layer upstream of the vein occlusion, and more elongated vasMφs, which are located in and around the vessel wall of the occluded vein. Our experiments demonstrated a significant accumulation as soon as post-occlusion day 3 that remained elevated through day 7. The comparatively lower CD11b and F4/80 expression by RT-PCR at day 7 compared to day 3 in the occluded retina is therefore not the result of a decrease of MP numbers but likely the result of differences in the level of expression during Mφ differentiation and activation. Using a combination of traceable nucleotide EdU injections and liposome clodronate-induced depletion of circulating, we show that the accumulation of both parMφs and vasMφs is to a considerable extent due to the recruitment and infiltration of Mo-derived Mφs. Intravenous liposome clodronate injection decreases iba1^+^EdU^+^ cell density counted 3 days after BRVO without affecting the iba1^+^EdU^neg^ cell population. We controlled that intravenous injection of liposome clodronate does not modify the density of both vasMφs and parMφs in non-occluded animals (data not shown).

We observed a significant transient increase of Ccr2 mRNA, a cytokine receptor that is strongly expressed on classical CCR2+Mo but is quickly downregulated upon recruitment to the tissue [15] and Ccl2 mRNA, its predominant cytokine. Using wildtype and *CCR2*^*RFP/RFP*^ mice, we demonstrate that CCR2+Mo recruitment does indeed participate in the Mφ infiltration after BRVO. Surprisingly, Ccr2 deficiency very strongly blunted the accumulation of parMφs but had no effect on vasMφs.

The observation that vasMφs can be strongly inhibited by Mo depletion, but not by Ccr2 ablation, strongly suggests that the vasMφs were recruited through a CCR2-independent mechanism. Non-classical monocytes that do not express Ccr2 and that are involved in other pathologies [11, 20] would seem a likely candidate. Indeed, a subgroup of non-classical Mo expressing high levels of the chemokine receptor CXCR4 has previously been shown to be angio-protective [21, 22] as it produces high level of VEGF [20] and specifically accumulates in the perivascular area in diseases such as autoimmune encephalomyelitis (EAE) [23].

Similarly, our analysis of endothelial cell apoptosis 3 days post-BRVO in control liposome and liposome clodronate-treated mice, and wildtype and *Ccr2*^*RFP/RFP*^ mice clearly shows that the inhibition of vasMφs recruitment, but not parMφs, leads to a dramatic increase of EC apoptosis in the occluded vein.

## Conclusion

Taken together, our study demonstrates for the first time an important role of blood-derived vasMφs in the vessel remodeling after BRVO. Our findings show a vasoprotective effect of blood-derived vasMφs that should be taken into consideration in the development of future therapies.

## Data Availability

All data generated or analyzed during this study are included in this published article. The datasets used and/or analyzed during the current study are also available from the corresponding author on reasonable request.

## Abbreviations

BRVO: Branch retinal vein occlusion
Ccl2: C-C chemokine ligand 2
Ccr2: C-C chemokine receptor type 2
ColIV: Collagen IV
EC: Endothelial cell
EdU: 5-Ethynyl-2′-deoxyuridine
Iba1: Ionized calcium-binding adapter molecule 1
Icam-1: Intracellular adhesion molecule 1
iMφs: Infiltrated macrophages
lipo: Control liposome-treated
lipo-clo: Liposome clodronate-treated
MC: Microglia
Mos: Monocytes
Mφs: Macrophages
parMφs: Parenchymal macrophages
rMφs: Resident macrophages
TUNEL: Terminal deoxynucleotidyl tranferase dUTP nick end labeling
vasMφs: Perivascular macrophages
